# Notes and News

**Published:** 1902-09-06

**Authors:** 


### Notes and News.

His Royal Highness the Prince of Wales and the Executive Committee of King Edward's Hospital Fund for London announce that they have received from Lord Mount Stephen and Lord Strathcona a munificent endowment which will produce upwards of £16,000 annually for the Fund. Lord Mount Stephen and Lord Strathcona have before shown magnificent generosity in the cause of the sick poor. In 1886 they built a hospital in Montreal at a cost of £200,000, and afterwards endowed it to commemorate Queen Victoria's Jubilee, this time devoting £160,000 for the purpose.

We regret that the word "Montrose" was printed instead of "Aberdeen" on page 387 of the Workshop section of our issue of August 30th.

There has been a good deal of scarlet fever round Bromsgrove and Redditch lately. There are upwards of 40 cases in the local isolation hospital, where the enteric wards have had to be requisitioned for their accommodation.

Some time ago a friend of the Royal Sea Bathing Infirmary offered to give £500 to recoup a sum which had been withdrawn from the invested funds of the institution, provided a like sum was forthcoming from public subscription. The appeal proved successful, and the anonymous donor has now made another offer of £500 on the same terms.

Sir William Church presided over a meeting of friends and sympathisers with Dr. W. Law, last week, on the occasion of presenting him with a token of practical sympathy from 700 members of the medical profession, taking the form of a cheque for £500. Special thanks were accorded by the meeting to Dr. Paramore, who so ably advocated and organised the testimonial to Dr. Law.

Some time ago the management of the Worcester Infirmary closed a ward owing to lack of funds. The closing of the ward was intended to bring to the notice of the public the insufficient support they were affording to the institution. A feeling of regret has been aroused, and an offer has been received to assist to refloor the ward if the committee will open it once more. This they have now consented to do on January 1st.

An organisation entitled the International Central Committee for the Prevention of Tuberculosis has been established at Berlin.

A Consumptive wing for 12 patients has been added to the Newhills Cottage Hospital, Aberdeenshire. The architect is Mr. George Coutts, of Aberdeen.

The International Congress for the Improvement of the Condition of the Insane is sitting at Antwerp under the presidency of the Belgian Minister of Justice, M. Van den Heuvel. The delegates are representative of a large number of countries in Europe and in Asia.

A new wing has been opened at the Sea Bathing Infirmary at Scarborough. The additions include two wards with 11 beds in each, one with 9 beds, and two single rooms for isolation cases. A small chapel has also been erected. The cost has been about £2,000. Mr. C. Edeson is the architect.

The Managers and Governors of the West Bromwich Hospital are differing over a proposal to add a dental department to the hospital. It appears that the Managers arranged a department, and appointed an honorary dentist without consulting the Governors, who objected on account of the cost of up-keep, which was estimated to exceed £100 per annum. Sir Ernest Spencer proposed the establishment of the department and offered to defray the cost. The Governors feel, however, that they should have been consulted, and the legality of the action of the Board may yet be contested.

A difficulty has arisen at Farnborough by the refusal of the military authorities to receive members of the families of soldiers residing outside the camp into the Military Isolation Hospital. The local authorities hold that it is the duty of the military authorities to look after the soldiers' families, and so they also demur at receiving such patients. The military authorities hold that as they do not recognise marriages outside the strength, they have no responsibility, and if this attitude is tenable the local authorities should clearly act upon it, and thus prevent the spread of infection among both civilians and military.

The Shop Hours Acts, 1892–95, limit the employment in shops of young persons under the age of 18 years to 74 hours a week, including meal times. A staff of nine inspectors of whom three are females, enforce the Acts in London. During the year ending March 31st last, 127,502 inspections were made, 10,194 irregularities were reported, and 100 convictions, with penalties amounting to £148 were obtained. The Seats for Shop Assistants Act, 1899, has brought a measure of comfort and some degree of relief to many a weary woman and tired girl. In the county of London alone 19,614 shops are affected by the Act, where 46,492 females are employed. Mr. Spencer, head of the Public Control Department of the London County Council, states that "as a rule employers recognise the usefulness of the Act, and are perfectly willing to carry out its requirements."

